# Design of a stabilized non-glycosylated Pfs48/45 antigen enables a potent malaria transmission-blocking nanoparticle vaccine

**DOI:** 10.1038/s41541-023-00619-9

**Published:** 2023-02-18

**Authors:** Thayne H. Dickey, Richi Gupta, Holly McAleese, Tarik Ouahes, Sachy Orr-Gonzalez, Rui Ma, Olga Muratova, Nichole D. Salinas, Jen C. C. Hume, Lynn E. Lambert, Patrick E. Duffy, Niraj H. Tolia

**Affiliations:** 1 Host-Pathogen Interactions and Structural Vaccinology Section, Laboratory of Malaria Immunology and Vaccinology, National Institute of Allergy and Infectious Diseases, National Institutes of Health (NIH), Bethesda, MD USA; 2 Vaccine Development Unit, Laboratory of Malaria Immunology and Vaccinology, National Institute of Allergy and Infectious Diseases, National Institutes of Health (NIH), Bethesda, MD USA; 3 Pathogenesis and Immunity Section, Laboratory of Malaria Immunology and Vaccinology, National Institute of Allergy and Infectious Diseases, National Institutes of Health (NIH), Bethesda, MD USA

**Keywords:** Protein vaccines, Malaria, Protein vaccines

## Abstract

A malaria vaccine that blocks parasite transmission from human to mosquito would be a powerful method of disrupting the parasite lifecycle and reducing the incidence of disease in humans. Pfs48/45 is a promising antigen in development as a transmission blocking vaccine (TBV) against the deadliest malaria parasite *Plasmodium falciparum*. The third domain of Pfs48/45 (D3) is an established TBV candidate, but production challenges have hampered development. For example, to date, a non-native N-glycan is required to stabilize the domain when produced in eukaryotic systems. Here, we implement a SPEEDesign computational design and in vitro screening pipeline that retains the potent transmission blocking epitope in Pfs48/45 while creating a stabilized non-glycosylated Pfs48/45 D3 antigen with improved characteristics for vaccine manufacture. This antigen can be genetically fused to a self-assembling single-component nanoparticle, resulting in a vaccine that elicits potent transmission-reducing activity in rodents at low doses. The enhanced Pfs48/45 antigen enables many new and powerful approaches to TBV development, and this antigen design method can be broadly applied towards the design of other vaccine antigens and therapeutics without interfering glycans.

## Introduction

Vaccines that disrupt the *Plasmodium* parasite lifecycle would be powerful tools in combatting malaria. Progress towards malaria control has largely stalled in the last decade and has reversed in some endemic areas highlighting the need for durable interventions that include vaccines^1^. The recent approval of the partially effective RTS,S vaccine demonstrates both the utility of a vaccine in malaria control as well as the need for additional improvements in malaria vaccine development^2^. One strategy for improving malaria vaccines is to target additional stages of the parasite life cycle such as transmission from human to mosquito^3–5^.

The Pfs48/45 protein is a promising malaria transmission-blocking vaccine (TBV) candidate. Pfs48/45 is present on the surface of sexual stage parasites transferred from human to mosquito during a blood meal and in the mosquito prior to fertilization, with peak surface expression on gametes and zygotes. Pfs48/45 is required for efficient parasite fertilization and oocyst formation in the mosquito, and serum from animals immunized with Pfs48/45 blocks oocyst development^6–10^. Human infection elicits antibodies to Pfs48/45, suggesting vaccine-induced immunity could be boosted by natural infection^11^. The precise function of Pfs48/45 remains unknown, although an interaction with another TBV candidate, Pfs230, has been proposed^6^.

**Fig. 1:**
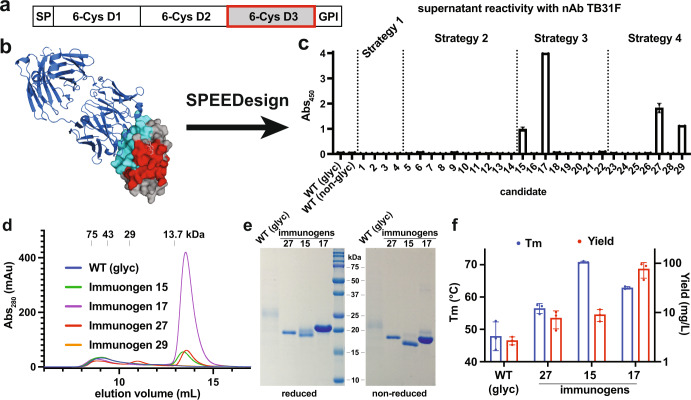
Design of a Pfs48/45 D3 antigen with improved yield and thermostability. **a** Overview of Pfs48/45 domain architecture. **b** Crystal structure of Pfs48/45 D3 (gray) in complex with the neutralizing Fab 85RF45.1 (blue) (PDB ID: 6E62). The predicted interface with D2 (red) was heavily designed while the neutralizing epitope (cyan) was retained. The N-glycan is shown in pink. **c** Reactivity of cell-free supernatant with neutralizing mAb TB31F for each transfected candidate. Values represent means and error bars indicate the standard deviation of technical replicate wells. **d** Size-exclusion chromatography profile of lead immunogens and WT Pfs48/45 after nickel purification. **e** SDS-PAGE analysis of purified lead immunogens. **f** Summary of melting temperature (T_m_) and purification yields for lead immunogens and WT Pfs48/45. Values represent mean and error bars indicate the standard deviation of biological replicates consisting of three independently expressed and purified protein batches.

Pfs48/45 contains three 6-cys domains (D1-3) and a C-terminal GPI anchor (Fig. 1a). Functions for each 6-cys domain have yet to be assigned, including the putative interaction site with Pfs230, limiting the ability to target biologically critical surfaces. Instead, monoclonal antibodies have been isolated and their binding has been mapped on the protein^12–14^. Antibody mapping studies found that antibodies to the N-terminal domain (D1) have poor transmission-reducing activity (TRA), antibodies to D2 have limited TRA, and antibodies to D3 can have potent TRA. The high-resolution crystal structures of a potently neutralizing antibody, 85RF45.1, and its humanized counterpart, TB31F, were solved in complex with D3, defining a potently neutralizing epitope^14,15^. This epitope contains only rare polymorphisms that do not substantially disrupt mAb binding, suggesting that a vaccine that elicits antibodies to this epitope could be broadly neutralizing.

The crystal structure of D3 also sheds light on stability and production challenges for Pfs48/45 that have limited vaccine development. The crystallized Pfs48/45 protein was produced in a eukaryotic expression system and expression required preservation of an NxS/T amino acid motif recognized by the oligosaccharyltransferase complex in these systems^15^. Indeed, an N-acetyl-glucosamine (NAG) was observed at position 303 in a loop of the protein, and NAG303 formed visible stabilizing contacts with a neighboring loop. There is no evidence that native Pfs48/45 is glycosylated and N-linked glycosylation in malaria proteins is generally limited, meaning that NAG303 may be a non-native mechanism of antigen stabilization. Consistent with this hypothesis, NAG303 lies at a surface predicted to be proximal to the N-terminal D2, which was not included in the crystallization construct. Together, these findings suggest that D3 requires stabilization for production, which can be achieved by glycosylation. However, the large glycans appended by eukaryotic systems other than *Plasmodium* do not reflect the native protein and may obscure neutralizing epitopes on the protein.

Stabilizer for Protein Expression and Epitope Design (SPEEDesign) is a ROSETTA-based computational and in vitro screening pipeline that designs antigens resulting in potent and durable vaccines^16^. The objectives of SPEEDesign are to improve the protective efficacy of a given antigen by: (1) focusing the immune response towards potently neutralizing antibody epitopes on the antigen; (2) reducing or eliminating immune responses to poorly neutralizing and/or immunodominant epitopes within the antigen; (3) optimizing the thermal stability of the antigen to increase its durability in vivo following immunization; (4) promoting conformational states of a protein antigen that may be hidden, for example by removing the glycan on pfs48/45 D3 that may mask neutralizing epitopes. SPEEDesign has proven successful in the development of improved SARS-CoV-2 antigens^16^.

Non-glycosylated Pfs48/45 D3 can be produced in bacterial systems, but the protein requires alternative means of stabilization and careful optimization to achieve proper disulfide bond formation. The protein can be expressed in *E. coli*, but requires fusion to maltose binding protein and co-expression of four chaperones^7^. The protein can also be produced in *L. lactis*, but requires fusion to Pfs230 or GLURP to produce high yields of properly folded protein^10,17^. The GLURP-fusion protein (R0.6C) is a promising pre-clinical vaccine candidate and several years of work have led to a reproducible purification process, however, the potential of a Pfs48/45 D3 vaccine has not been completely evaluated because the domain cannot be produced in isolation^18^. Here, we sought to create an easily produced stand-alone Pfs48/45 D3 antigen suitable for TBV development. We used a SPEEDesign computational design process to create immunogens suitable for expression in a eukaryotic system without requiring glycosylation. These immunogens can be expressed at yields much greater than the glycosylated wildtype (WT) protein, they have increased thermostability, and they retain the potent transmission-blocking epitope. Immunization of rats with these immunogens also elicited transmission-blocking antibodies. Further optimization of the immunogens resulted in a clinically relevant nanoparticle vaccine that elicits potent TRA in rats with two low-dose immunizations.

## Results

### Non-glycosylated immunogens have improved yield and thermostability, and retain the neutralizing epitope

SPEEDesign was used to create an enhanced pfs48/45 D3 immunogen (Fig. 1b). We sought to create a pfs48/45 D3 immunogen that could be expressed as a non-glycosylated stand-alone antigen in eukaryotic systems without any fusion partners that may detract from the immune response to pfs48/45. The standard SPEEDesign protocol was modified to include ablation of the glycosylation site during design.

SPEEDesign first requires a definition of the role of each amino acid within the protein, and consequently its mutability during the computational design process. The neutralizing epitope of mAb 85RF45.1 was retained by disallowing mutation during the computational design process. The residues predicted to contact the N-terminal domain 2 of pfs48/45, including the location of the stabilizing NAG303, were thoroughly searched during the design process to identify changes that would stabilize domain 3 upon extraction from the full-length protein (Supplementary Fig. 1). All remaining residues were allowed to sample a sequence space limited by energetic and evolutionary constraints.

SPEEDesign uses four different computational strategies that differ in their depth of design for each class of residue to generate 40,000 computational decoys. A clustering algorithm was then used to identify 29 candidates that sample a diverse sequence space (Fig. 1c). These candidates were screened in a mammalian expression system by capturing His-tagged immunogens from cell-free supernatant using a Ni-coated plate and measuring binding of the neutralizing mAb TB31F by enzyme-linked immunoassay (ELISA). Screening identified four candidates that had reactivity to TB31F greater than WT D3. All four candidates derive from strategies 3 and 4, which sample an intermediate sequence space that is less restrictive than strategy 1 and more confined than strategy 2 (see “Methods”).

Three lead immunogens (15, 17, and 27) could be expressed and purified as monomeric proteins with yields far greater than WT D3 (Fig. 1d–f). Mutation of the N-linked glycosylation sites in WT D3 resulted in no monomeric protein, and the glycosylated form had low yields of 2–3 mg/L, consistent with previous observations (Fig. 1f and Supplementary Fig. 2)^15^. Conversely, non-glycosylated immunogens 15 and 27 had yields of 9 and 8 mg/L, respectively, and immunogen 17 had an average yield of 77 mg/L, more than 25-fold higher than WT D3.

All three lead immunogens were more thermostable than WT D3 (Fig. 1f and Supplementary Fig. 3). Melting temperatures (T_m_) were measured by differential scanning fluorimetry (DSF) to determine if the enhancing mutations stabilize the designed antigens and fully compensate for the removal of the stabilizing glycan. Glycosylated WT D3 had a T_m_ of 48 °C, while the non-glycosylated designed immunogens had T_m_s 8–23 °C higher. Interestingly, the immunogen with the highest T_m_ (immunogen 15) was not the same as the immunogen with the highest yield (immunogen 17), suggesting these characteristics are not strictly correlated.

We used biolayer interferometry (BLI) to measure the integrity of the neutralizing epitope in a quantitative fashion (Supplementary Fig. 4 and Table 1). The neutralizing antibody TB31F bound all immunogens with sub-nanomolar affinity that was better than WT D3. These affinities suggest that the neutralizing epitope is unperturbed in all three lead immunogens, and that glycosylation of the WT protein may interfere slightly with the binding of neutralizing antibodies. Similarly, glycosylation of a WT vaccine antigen may mask neutralizing epitopes, supporting the need for a non-glycosylated immunogen.

**Table 1:** The neutralizing mAb TB31F binds lead immunogens 15, 17, and 27 stronger than WT D3.

	K_D_ (nM)	K_a_ (1/Ms) × 10^5^	K_dis_ (1/s) × 10^−4^	N
WT D3	2.5 ± 0.4	3.5 ± 0.7	8.2 ± 0.9	3
Immunogen 15	0.8 ± 0.06	11 ± 6	8.5 ± 0.4	3
Immunogen 17	0.69 ± 0.09	12 ± 6	8.0 ± 0.7	3
Immunogen 27	0.62 ± 0.05	13 ± 6	7.9 ± 0.4	3

Binding affinities and kinetic data determined by BLI.

### Immunogens elicit functional antibodies in rodents

**Fig. 2:**
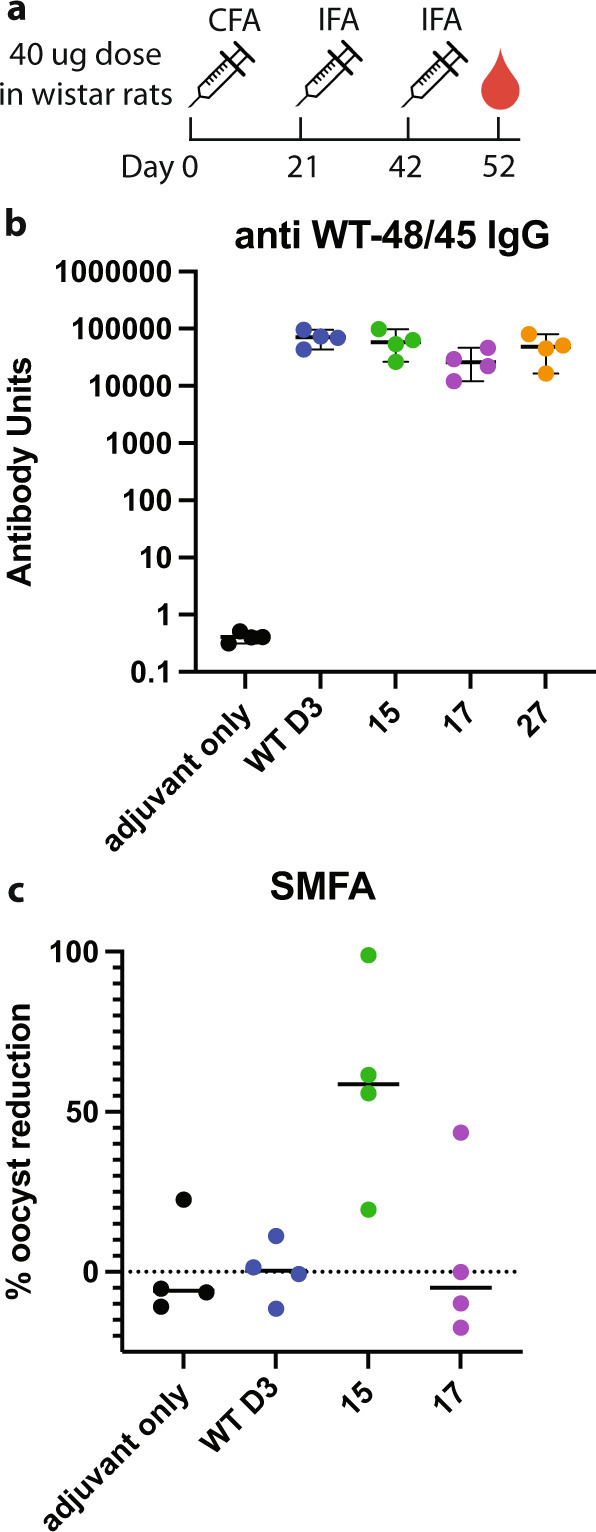
Immunization of rats with Pfs48/45 immunogens elicits TRA. **a** Immunization and blood draw regimen. **b** IgG antibody titers to WT Pfs48/45 D3. Median values and 95% confidence intervals are displayed. **c** TRA of serum from immunized rats with median values displayed. Dashed line indicates 0% TRA relative to adjuvant only serum.

We immunized rats with the three lead immunogens and the control antigen WT D3 to determine if the immunogens could elicit an improved immune response (Fig. 2a). We immunized the rats with three doses of 40 µg antigen adjuvanted in Freund’s adjuvant (CFA/IFA). All four antigens elicited high titers of IgG that recognized WT D3 (Fig. 2b). The immunogens elicited slightly lower titers than WT D3 consistent with the loss of non-neutralizing epitopes outside of the TB31F epitope.

We hypothesized that the immunogens would elicit higher levels of functional antibodies, despite the lower overall antibody titers, because the immune response would be focused to the neutralizing epitope. Indeed, pooled serum from rats immunized with immunogens 15 and 17 contained higher transmission-reducing activity (TRA) than WT D3 in a standard membrane feeding assay (SMFA) (Supplementary Fig. 5). Immunogen 27 elicited no detectable TRA, potentially due to its lower thermostability. We further analyzed the serum from individual animals and found that immunogen 15 elicited moderate TRA in three of the four animals and immunogen 17 elicited moderate TRA in one animal (Fig. 2c). WT D3, however, elicited very little TRA in all four animals, suggesting that the immunogens improved the functional antibody response in rats.

### Optimization of immunogens through reversion to WT sequence

**Fig. 3:**
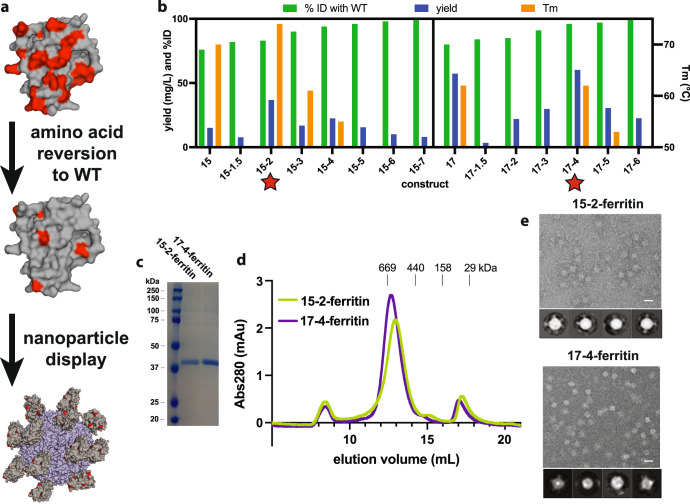
Creation of nanoparticles displaying optimized immunogens. **a** Antigen optimization process first reverting designed amino acids (red) back to WT identity (gray), followed by display on a 24-copy ferritin nanoparticle (blue). **b** Screening of optimized immunogen monomers. Nickel purification yields are reported for all constructs. T_m_ was measured for those constructs with sufficient yields. Immunogens 15-2 and 17-4 were identified as optimized leads for nanoparticle display. **c** SDS-PAGE analysis of purified immunogens fused with ferritin. **d** Size-exclusion chromatogram of purified particles after freeze thaw, indicating formation of a stable particle. **e** Negative-stain EM micrographs of purified nanoparticles displaying optimized immunogens. Scale bars are 20 nm. The 2D class averages are shown below each micrograph.

The partial TRA conferred by immunogens 15 and 17 provided a starting point for further optimization of these immunogens (Fig. 3). mAb 85RF45.1 (and the humanized version TB31F) is the only antibody for which structural information exists, meaning that we lack a comprehensive epitope map of pfs48/45 D3. Therefore, mutations in immunogens 15 and 17 may perturb undiscovered neutralizing epitopes, limiting the efficacy of these vaccine antigens. To restore potential neutralizing epitopes, we iteratively reverted amino acids to their WT identity without restoring the N-glycosylation site.

We used a computational strategy for amino acid reversion that prioritized changes based on predicted thermodynamic stability in ROSETTA. Those amino acid reversions that had beneficial or neutral energetic effects were made first and additional designs were created with increasingly energetically unfavorable reversions added. For example, 17-6 has more amino acid reversions and is more homologous to WT D3 than 17-2, but 17-6 is predicted to be less energetically stable than 17-2.

We expressed the reversion candidates for immunogens 15 and 17 in mammalian cell culture and identified several that had purification yields as good or better than the parent immunogen (Fig. 3b). Most of these candidates could be purified as well-behaved monomeric proteins with 15-2 and 17-4 having the highest yields (Supplementary Fig. 6a and b). We further down-selected the reversion candidates based on T_m_, resulting in two optimized leads: immunogens 15-2 and 17-4 (Fig. 3b and Supplementary Fig. 6c and d). Immunogens 15-2 and 17-4 have yields and thermostabilities at least as good as their parent antigens, which were already improved over WT D3. Furthermore, immunogens 15-2 and 17-4 have 83% and 95% identity to WT D3, compared to the 76% and 79% identities of their respective parent antigens. Thus, immunogens 15-2 and 17-4 retain the benefits of the parent antigens over WT D3 and they may have undiscovered neutralizing epitopes restored.

### Enhancing immunogenicity of the antigens through nanoparticle display

We sought to further improve the efficacy of our immunogens via nanoparticle display (Fig. 3a). Presentation of an antigen on a protein nanoparticle can dramatically increase functional antibody titers, especially when the antigen is small and poorly immunogenic, like pfs48/45 D3^19^. While our immunogen is likely compatible with many nanoparticle platforms, we opted for a genetic fusion to *H. pylori* ferritin to create a single-component self-assembling particle that can be easily manufactured and has an established record of safety in human clinical trials^20^.

Immunogens 15-2 and 17-4 could be clearly expressed as stable intact ferritin fusions. In contrast, WT D3-ferritin exhibited no visible expression (Supplementary Fig. 7). A single purification step using size-exclusion chromatography yielded pure fusion protein that eluted at a size consistent with the formation of a 24-copy nanoparticle (Fig. 3c, d). Negative-stain electron microscopy images revealed pure nanoparticles and the 2D class averages showed visible antigen displayed on the exterior (Fig. 3e). In summary, antigen optimization resulted in 24-copy single-component nanoparticles displaying pfs48/45 D3 immunogens 83% and 95% identical to the WT sequence (Supplementary Fig. 10).

### Optimized immunogen nanoparticles elicit significantly higher TRA than WT D3

**Fig. 4:**
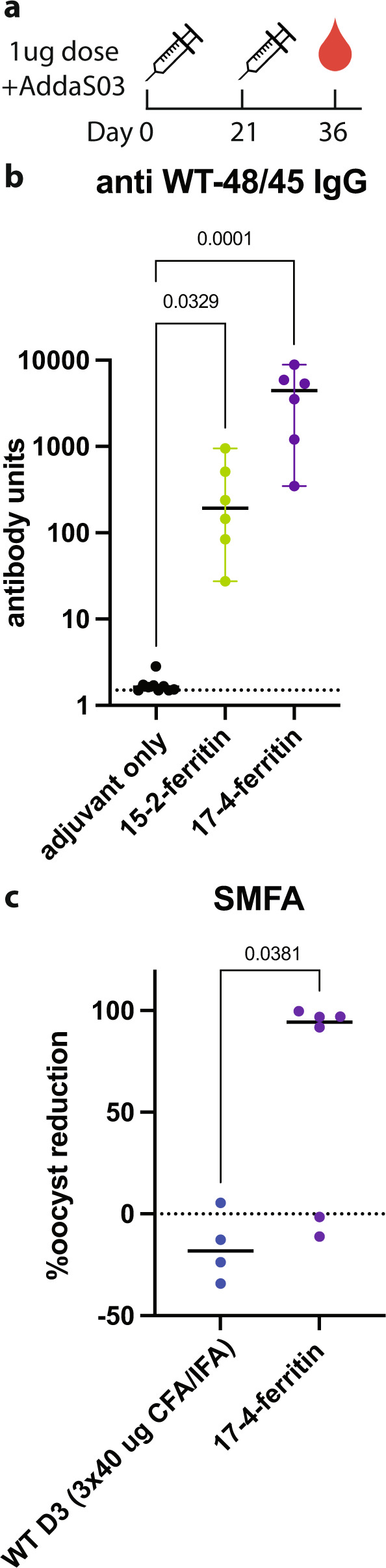
Two low-dose immunizations with a Pfs48/45 D3 immunogen nanoparticle elicits high TRA. **a** Immunization and blood draw schedule for Wistar rats. **b** IgG antibody titers to WT Pfs48/45 D3. Medians and 95% confidence intervals are displayed. Dashed line indicates the limit of detection. *P*-values displayed were calculated using a Kruskal–Wallis test followed by a Dunn’s comparison to adjuvant only, corrected for multiple comparisons. **c** TRA of serum from immunized rats with median values indicated. Dashed line indicates 0% TRA relative to adjuvant only serum. *P*-value displayed was calculated using a two-tailed Mann–Whitney test.

Rats were immunized with the optimized immunogen nanoparticles to determine if they elicited functional antibodies (Fig. 4). To better scrutinize their suitability for clinical development, we administered two low doses of antigen (1 μg) adjuvanted in AddaS03, a mimic of the AS03 adjuvant approved for human use (Fig. 4a). Serum was analyzed 14 days after the second immunization, and we found that both particles elicited high titers of IgG that recognized WT D3 (Fig. 4b). However, the titers elicited by 17-4-ferritin were higher than for 15-2-ferritin. Pooled serum from the group immunized with 17-4-ferritin also exhibited high levels of TRA, while 15-2-ferritin elicited no TRA (Supplementary Fig. 8). We further analyzed serum from the individual animals immunized with 17-4-ferritin to find that serum from 4/6 animals had very high TRA > 90%, indicating a very potent functional immune response. The two animals in the 17-4-ferritin group that had low TRA also had the lowest antibody titers, suggesting that increasing titers through simple steps like higher dosing or more immunizations may lead to a complete response. In summary, the TRA elicited by two low doses of 17-4-ferritin in a human-grade adjuvant is significantly higher than that elicited by three high doses of WT D3 adjuvanted in CFA/IFA (Fig. 4c).

## Discussion

We have created an effective and potent stand-alone Pfs48/45 immunogen through structure-based design. Rats immunized with a low dose of antigen formulated in a clinically relevant adjuvant produce high titers of transmission-reducing antibodies. The success of this vaccine, and the SPEEDesign process, can be attributed to an increase in the transmission-blocking quality of the antibody response rather than a simple increase in anti-Pfs48/45 antibody titers (Supplementary Fig. 9). For example, immunization with WT D3 produces very high antibody titers, but no TRA (Fig. 2). Immunogen 15 produces slightly lower titers than WT D3, but higher TRA, suggesting the quality of the antibody response is improved. Similarly, 17-4-ferritin elicits higher TRA than the parent immunogen 17, but with lower antibody titers. The quality of the antibody response can be quantified by expressing TRA normalized to the level of Pfs48/45 antibodies (i.e., calculating the ratio of %TRA to the titer of Pfs48/45 antibodies measured by ELISA), which clearly indicates that the quality of the antibody response improves through the design process (Supplementary Fig. 9).

There are several reasons that the design process might improve the quality of the antibody response. First, removal of the large non-native glycan could expose neutralizing epitopes, increasing the antibody response to these epitopes in immunized animals. This conclusion is supported by the fact that the neutralizing mAb TB31F binds more tightly to the immunogens than WT D3. Second, the increased thermostability of the immunogens may increase their duration and conformational stability in the body of immunized animals, particularly in germinal centers where affinity maturation can continue for weeks. Third, redesign of the exposed D2/D3 interaction surface on D3 may reduce immunogenicity to non-natural surfaces created upon extraction of D3 from the full-length Pfs48/45. Fourth, the amino acid reversion process may restore undiscovered neutralizing epitopes. Fifth, nanoparticle display may selectively present neutralizing epitopes and/or affect the B cell maturation process in a way that increases neutralizing antibody activity. These factors are not mutually exclusive and there are likely several factors that contribute to the improved antibody response elicited by the designed immunogens.

The immunogens presented here enable a wide array of vaccination strategies that were previously impossible. The creation of a Pfs48/45 antigen without the NxS/T glycosylation motif now allows production of the antigen in any organism without the risk of a non-native glycan obscuring functional epitopes. This means the antigen can be faithfully produced in human cells, enabling the use of viral vectored and nucleic acid vaccine platforms. Widely used recombinant platforms like yeast, insect cells, and CHO cells can also be used to produce these antigens without the addition of large branched glycans completely unlike those found in the malaria parasite. These recombinant platforms are particularly enticing because they are well-suited for producing proteins with complex disulfide patterns like Pfs48/45. Furthermore, these recombinant platforms allow co-expression or fusion of many different proteins such as the nanoparticle carrier we have showcased here.

One limitation of this study is that two rats did not produce detectable levels of transmission-reducing antibodies. We expect that this limitation can be readily overcome by optimization of dose, formulation, and vaccination schedule leading to a complete response. Furthermore, the SMFA assay includes human complement, which can be fixed by antibodies to increase transmission-reducing activity. However, human complement is not readily fixed by rat antibodies, and we therefore are not evaluating one effector function of the immune response. It is likely that the SMFA activity of this vaccine will be augmented in species like humans, primates, and rabbits where antibodies more efficiently fix human complement.

This study is limited to the analysis of TRA against a homologous parasite strain, but there is evidence that our Pfs48/45 immunogen could elicit strain-transcending blocking activity. Early studies indicated that Pfs48/45 was a promising TBV candidate because of its high sequence conservation and the ability of transmission-blocking mAbs to cross-react with diverse parasite isolates^21–24^. The moderate level of sequence variation is comparable to other *Plasmodium* 6-cys proteins^25^. More recently, high-throughput and systematic characterization of parasite isolates have corroborated these early studies. In the Pf3k database of >2500 *P. falciparum* samples, there are only 9 polymorphic positions within Pfs48/45 D3 and other studies have identified three additional polymorphic positions^26–28^. Only three of these 12 polymorphisms lie within the 85RF45.1/TB31F epitope and they occur at <0.1% frequency in the Pf3k database^28^. Two of these polymorphisms do not perturb 85RF45.1 binding, and while the third has not been tested, there are likely to be conserved neutralizing epitopes on the protein^14,15^. mAb 85RF45.1 has TRA against diverse parasite isolates, consistent with earlier work showing cross-reactive binding to the same epitope^24,29^. Our immunogens retain the 85RF45.1/TB31F epitope and surrounding residues, suggesting they would elicit strain-transcending antibodies. Strain-transcending responses would need to be evaluated in future studies of our immunogen in higher organisms, such as NHPs and humans, because the nature of the antibody response can be species-specific.

The design process presented here is generalizable and will be useful in creating enhanced non-glycosylated antigens for various pathogens. Many different pathogens have glycosylation patterns that are not recapitulated by recombinant production platforms and that may result in occluded neutralizing epitopes. Simple mutation of the NxS/T motif often destabilizes an antigen, as shown for Pfs48/45, but SPEEDesign can counteract this destabilization to create an effective antigen suitable for recombinant production. This design method is especially applicable to antigens from parasites that have unique glycosylation pathways, such as Plasmodium, Toxoplasma, Leishmania, and Trypanosoma^30–33^. RTS,S is the only parasite vaccine approved for human use, but SPEEDesign will facilitate the production of many new parasite antigens and vaccine candidates where the prerequisite information is available^3,34–48^. Vaccines against viruses could also benefit from SPEEDesign stabilization of de-glycosylated antigens. Viruses often use glycans to shield neutralizing epitopes from antibodies, and glycan removal may elicit an antibody response to neutralizing epitopes that would otherwise be occluded^49–51^. For example, arenaviruses with fewer N-linked glycosylation sites elicit higher titers of neutralizing antibodies, suggesting that additional removal of glycans through SPEEDesign will produce even higher neutralizing antibody titers^52^.

The numerous benefits of a non-glycosylated antigen described above are some of the aspects that distinguish these SPEEDesign immunogens from another recently reported stabilized Pfs48/45-D3 nanoparticle^53^. Mcleod et al. created thermostabilized immunogens that could be displayed on nanoparticles and similarly elicited TRA much greater than WT D3, supporting the notion that antigen stabilization is a powerful method to improve vaccine efficacy. However, the design methods used in each study are unique, and none of the stabilizing mutations are similar to those in 17-4 (Supplementary Fig. 10). The antigen created by Mcleod et al. remains glycosylated and has purification yields approximately 10-fold lower than 17-4. In conjunction with our uses of SPEEDesign^16^, the current studies underscore the benefits of immunogen design in vaccine development.

Non-glycosylated proteins also have manufacturing and safety benefits over glycoproteins. The precise glycan structure and composition on a glycoprotein depends on production platform and can be influenced by subtle changes in growth conditions^54–56^. This can lead to heterogeneity within a batch of glycoprotein and variation between batches. Alternatively, the composition of a non-glycosylated antigen is much more homogenous and reproducible. In addition, glycoproteins can cause unwanted immune reactions if they contain non-human glycans, as was observed in the severe hypersensitivity reaction to a murine glycan on cetuximab^57^. Non-glycosylated proteins would not be at risk for this problem, making SPEEDesign potentially applicable to therapeutic proteins, as well as vaccine antigens, as demonstrated by the development of a potent transmission-blocking vaccine for malaria.

## Methods

### Prediction of the D3 interaction surface with D2

A model of the Pfs48/45 D2/D3 interaction surface was created by aligning the structures of Pfs48/45-D3 and the C-terminal 6-cys domains of P12 and P41. P12 and P41 are tandem 6-Cys domains with similar D2/D3 interaction interfaces, suggesting this interface is relatively conserved in tandem 6-Cys domains. Residues of Pfs48/45-D3 that contact either N-terminal 6-Cys domain of P12 or P41 were then defined as a putative interface for design. Contact residues, and other interfaces, were defined as those that have a >1 Å change in solvent-accessible surface area upon removal of the interacting partner, and calculations were performed in PyMOL^58^.

### SPEEDesign computational steps

The SPEEDesign pipeline categorizes each residue as fixed, intermediate, or deep search to define the amino acids identities sampled at each position during the computational design process^16^. Pfs48/45-D3 residues that form the interface with the neutralizing mAb 85RF45.1 were defined as fixed, leaving them unchanged through the design process^14,15^.

Residues exposed upon the extraction of a domain from a larger protein require are defined as deep search in SPEEDesign^16^. Since these residues are not exposed in homologous proteins, conservation or evolutionary-based design principles may not prove sufficient to redesign these new non-natural surfaces. For Pfs48/45-D3, we defined these newly exposed residues by modeling the D2/D3 interface (see above). We also defined the NxS/T motif and all residues that contact the glycan as deep search, allowing them to sample all amino acids, except cysteine.

All other residues were defined as intermediate. These residues were allowed to vary to a limited extent that is driven by conservation and evolutionary analysis of similar protein sequences to identify potential amino acid changes^16^.

SPEEDesign uses four ROSETTA design strategies. All ROSETTA strategies left fixed residues unchanged to preserve neutralizing epitopes, and each strategy differs in the amino acid changes allowed for the intermediate and deep search categories of residues. In strategy 1, intermediate residues were unchanged and all amino acids except cysteine were allowed at deep search positions. In strategy 2, all amino acids were allowed at deep search positions, and intermediate positions were allowed to sample amino acids found in proteins with similar sequences (evolutionary constraints). Evolutionary constraints in strategy 2 were defined by creating a position-specific scoring matrix (PSSM) using PSI-BLAST and including variant amino acids in the ROSETTA design process if they had scores >0 in the PSSM^59^. As a result, strategy 2 sampled a very large sequence space. This sequence space was constrained in strategy 3 by disallowing amino acid changes that are energetically unfavorable when made individually (energetic constraints). The energetic constraints in strategy 3 were imposed by using the ROSETTA FilterScan module, which estimates the energetic effect of individual amino acid changes^60^. Amino acid changes from the PSSM were disallowed if they had energetic penalties (delta_filters) >0.5 Rosetta energy units (R.e.u.). Strategy 4 placed the same evolutionary and energetic constraints on the intermediate residues as strategy 3 but with a more stringent energetic cutoff of −0.45 R.e.u. In addition, strategy 4 ignored all energetic and evolutionary restraints for deep search residues, allowing them to sample all amino acids. These amino acid constraints were used to create a ResFile for each strategy that was used by the FastDesign module in ROSETTA.

For each computational strategy, decoys with scores in the 95th percentile were clustered by sequence similarity using CD-HIT and the top scoring decoy form each cluster was selected as a representative sequence^61^. The number of clusters was selected based on the sequence diversity produced in each computational strategy.

### SPEEDesign in vitro screening

Synthetic DNA coding for secreted immunogens was cloned (GenScript) into a customized pHL-sec expression plasmid. pHL-sec was a gift from Edith Yvonne Jones (Addgene plasmid # 99845; http://n2t.net/addgene:99845; RRID:Addgene_99845)^62^. Plasmid was transfected into human expi293F cells and grown in a 96-well plate according to manufacturer instructions (ThermoFisher Scientific). Cell-free supernatant was harvested after 4 days of expression.

Cell-free supernatant was diluted 1:3 in phosphate-buffered saline (137 mM NaCl, 2.7 mM KCl, 10 mM Na_2_HPO_4_, 1.8 mM KH_2_PO_4_) with 0.05% Tween20 (PBST) + 2% bovine serum albumin (BSA) and added to nickel-nitrilotriacetic acid (Ni-NTA) HisSorb Plates (Qiagen) to capture His-tagged immunogens. After incubation for 1 h at room temperature, plates were washed three times with PBST. Neutralizing epitopes were probed using a TB31F-Fc (IgG1) protein (0.05 µg/200 µL/well). After incubation for 1 h at room temperature, plates were washed three times with PBST and 200 µl 1:5000 peroxidase-conjugated anti-human IgG was added (Jackson ImmunoResearch Laboratories, Inc., Cat. # 109-035-098). Plates were incubated 30 min at room temperature and washed three times with PBST. Finally, 70 µl Tetramethylbenzidine (TMB) (MilliporeSigma) was added and incubated 2 min at room temperature before quenching with 70 µl 0.16 M H2SO4. Absorbance at 450 nm was measured using a Biotek Synergy H1 plate reader.

### Amino acid reversion to native sequence

Amino acids that were changed in the original immunogens 15 and 17 were individually reverted to their native identity. The energetic effect of this reversion was calculated using ROSETTA filterscan mover^60^. The suffix of the reversion construct indicates the energetic penalty that was allowed during the reversion process. For example, 15-2 contains all individual reversion mutations with a delta filter threshold of 2 in filterscan, and 17-4 contains all mutations with a delta filter threshold of 4 in filterscan.

### Immunogen expression, purification, and calculation of yields

Recombinant immunogens were expressed in expi293F cells, as described for SPEEDesign in vitro screening above. Cell-free supernatant was harvested 4 days after transfection and His-tagged antigens were purified by gravity chromatography using Ni Sepharose excel resin according to manufacturer instructions (Cytiva). Antigens were further purified by size-exclusion chromatography using a Superdex 75 Increase 10/300 GL column equilibrated in 1x PBS. Fractions corresponding to monomeric immunogen were pooled, snap frozen in liquid nitrogen, and stored at −80 °C.

Transfection, expression, and purification of original designs were performed in triplicate on three separate days to calculate immunogen purification yields. Each replicate consisted of a 30 mL culture, and yields were calculated by integrating the area under the monomeric peak on the Abs_280_ chromatogram during size-exclusion chromatography. These yields closely matched yields calculated from pooled fractions. Purification yields for reversion mutants was calculated after nickel purification by measuring the Abs_280_ of eluant. Extinction coefficients were calculated using the ExPASy ProtParam tool^63^. Sample purity was assessed by SDS-PAGE (Supplementary Figure 11).

### TB31F cloning, expression, and purification

TB31F was created by fusing the variable regions to the human IGHG*01 or IGLC2*02 constant regions and cloning into the pHL-sec plasmid (GenScript). Heavy and light chain plasmids were mixed in equal amounts and transfected into expi293F cells according to manufacturer instructions and cell-free supernatant was harvested after 4 days of expression.

Cell-free supernatant was batch incubated with protein A agarose resin (GoldBio) for 1 h at room temperature. Resin was collected and washed with 10 column volumes (CV) protein A IgG binding buffer (ThermoFisher Scientific). Protein was eluted with 10 CV IgG elution buffer (ThermoFisher Scientific) and neutralized with 1 CV 1 M Tris pH 9.0. Antibody was concentrated and buffer exchanged into PBS using an Amicon centrifugal filter (MilliporeSigma).

### Nanoparticle expression and purification

Nanoparticles were created by genetically fusing ferritin to the C-terminus of Pfs48/45 immunogens. There is a GSGGGG linker between the immunogens and ferritin. The ferritin construct is an engineered construct of fused bullfrog (*Rana catesbeiana*) and *H. pylori* ferritin^64^. Residues 2-9 (Uniprot P07797) of *Rana catesbeiana* ferritin with an N8Q mutation were fused to residues 3-167 (UniProt: Q9ZLI1) with I7E and N19Q mutations. All constructs were created without additional purification or solubility tags.

Proteins were expressed in expi293F cells, as described above. Cell-free supernatant was harvested after 4 days of expression at 37 °C and concentrated using 100,000 kDa molecular weight cutoff Amicon centrifugal filter units. Concentrated supernatant was purified by size-exclusion chromatography using a Superose 6 Increase 10/300 GL column (Cytiva) equilibrated in 1x PBS. Fractions corresponding to assembled nanoparticle were pooled, snap frozen in liquid nitrogen, and stored at −80 °C.

### Differential scanning fluorimetry

DSF was performed using the Protein Thermal Shift Dye Kit according to manufacturer instructions (ThermoFisher Scientific). Final reactions contained 0.4 mg/mL purified immunogen, 1x Protein Thermal Shift buffer, 1x Thermal Shift Dye, and 0.63x PBS. Fluorescence was monitored using a 7500 Fast Real-Time PCR system (ThermoFisher Scientific) as the temperature was increased from 25 °C to 95 °C at a ramp rate of 1%. Melting temperature (T_m_) was calculated using the Boltzmann method. DSF reactions were performed in technical quadruplicate on each plate and in biological triplicate using three different protein preps on 3 separate days for original designs. Reversion mutants were analyzed as a single biological replicate. Technical replicates were averaged to calculate the T_m_ for a biological replicate, and biological replicates were averaged to calculate the reported T_m_.

### Biolayer interferometry

The binding affinity of purified immunogens to TB31F was measured using a kinetic BLI assay using an Octet-Red96e (Sartorius)^16^. TB31F was buffer exchanged into HBS-EP buffer (10 mM Na-HEPES pH 7.4, 150 mM NaCl, 3 mM EDTA, 0.005% v/v P20 surfactant) using Zeba spin desalting columns (ThermoFisher Scientific). TB31F at 10 nM was loaded onto Anti-hIgG Fc Capture (AHC) biosensors (Sartorius) over the course of 300 s, until reaching a signal of ~0.6 nm. BLI pins were then immersed in immunogens 2-fold serially diluted in HBS-EP buffer (30 nM to 0.469 nM). After 300 s, pins were immersed in HBS-EP buffer to measure dissociation. Association rate (k_a_), dissociation rate (k_dis_), and dissociation constant (K_D_) were globally fit using a 1:1 binding model in Data Analysis HT 12.0 (Sartorius). Three independent protein preps (biological replicates) were each measured in technical triplicate. Values reported are the average and standard deviation between biological replicates.

### Negative-stain electron microscopy

Purified nanoparticles (0.01 mg/ml) were adsorbed on a glow discharged 300 mesh carbon-coated copper grids (EMD Science) for 30 s followed by NanoW (Nanoprobes) staining. Raw Micrographs were recorded in Thermo Scientific Tecnai T20 microscope equipped with a charge-coupled device (CCD) camera. Particles were auto-picked using Gautomatch (http://www.mrc-lmb.cam.ac.uk/kzhang/) and 2D classes were generated using RELION 3.0^65^.

### Rat immunizations

For CFA/IFA studies, immunizations were performed by Noble Life Sciences in an AAALAC-accredited facility under the guidelines and approval of the Institutional Animal Care and Use Committee (IACUC) approved, and under OLAW assured conditions. Male wistar rats (Envigo) weighing 225–250 g were immunized by subcutaneous injection, delivering 40 μg antigen per animal in a 100 μl volume. Monomeric antigens were purified, as described above, snap frozen in liquid nitrogen, and stored at −80 °C. On the day of immunization, antigen was thawed and formulated as a 1:1 ratio in Complete Freund’s Adjuvant (MilliporeSigma) on day 0 and Incomplete Freund’s Adjuvant (MilliporeSigma) on days 21 and 42. Blood was collected on indicated days, and serum was separated and stored at −80 °C.

AddaSO3 immunizations were performed in an AAALAC-accredited facility under the guidelines and approval of the Institutional Animal Care and Use Committee (IACUC) at the National Institutes of Health. Nine-week-old female CD rats (Charles River Laboratories) were immunized with 1 μg antigen in a 100 μl volume by subcutaneous injections given on days 0 and 21. Nanoparticle antigens were purified, as described above, snap frozen in liquid nitrogen, and stored at −80 °C. On the day of immunization, antigen was thawed and formulated as a 1:1 ratio in AddaS03, according to manufacturer recommendations (Invivogen). Blood was collected on indicated days, and serum was separated and stored at −80 °C.

### Serum antibody titer ELISAs

Nunc MaxiSorp plates (ThermoFisher Scientific) were coated with 100 µl 0.005 mg/mL purified WT Pfs48/45-D3 diluted in 50 mM Na-carbonate pH 9.5. Plates were incubated overnight at 4 °C then washed three times with PBST. Plates were blocked 1 h at room temperature with 2% BSA in PBST then washed three times with PBST. Serum was diluted in 2% BSA in PBST and 100 µl was added to each well. After 1 h incubation at room temperature, plates were washed three times with PBST and 200 µl 1:10,000 peroxidase-conjugated anti-rat IgG (Jackson ImmunoResearch Laboratories, Inc., Cat# 112-035-071) was added. Plates were incubated 30 min at room temperature and washed three times with PBST. Finally, 70 µl Tetramethylbenzidine (TMB) (MilliporeSigma) was added and incubated 20 min at room temperature before quenching with 70 µl 0.16 M H2SO4. Absorbance at 450 nm was measured using a Biotek Synergy H1 plate reader.

Pooled serum from rats immunized with WT D3 in CFA/IFA was used as a standard curve on each plate to calculate the antibody titers of individual animals in all groups. One antibody unit (AU) was defined as the dilution of the standard serum required to achieve an Abs_450_ value of 1. Each plate included triplicate 2-fold serial dilutions of the standard serum from 20 to 0.01 AU. Serum from each animal was diluted such that the Abs_450_ fell in the informative portion of the standard curve between 0.1 and 2.0. The Abs_450_ values for the standard curve were fit to a 4-parameter logistic curve, which was used to convert Abs values to AU for each individual animal. AU values for each individual animal were measured in triplicate on separate plates and the average was reported.

### Standard membrane feeding assay (SMFA)

Functional activity of immune sera was assayed by an ex vivo standard membrane feeding assay (SMFA) in terms of their transmission-reducing activity (TRA, reduction in mosquito infection intensity)^36,66^. In vitro 14–16-day-old gametocyte culture of *P. falciparum* (NF54 line) was diluted with washed O + red blood cells (RBCs) from a malaria naive donor and AB + human naive serum pool to achieve 0.12 ± 0.05% concentration of Stage V gametocytes and 50% hematocrit. Test samples, containing 30 μL of test serum and 30 μL of naive human AB + serum pool (60 µL total volume each), were prepared in advance. Each test sample (60 µL) was mixed with 200 µL aliquot of diluted culture and immediately fed to pre-starved (~24 h) 3–8-day-old *Anopheles stephensi* (Nijmegen strain) mosquitoes through a membrane feeding device connected to a 40 °C circulating water bath. After feeding, mosquitoes were maintained at 26 °C and 80% humidity conditions to allow for the development of parasites. On day 8 mosquito midguts were dissected and stained with 0.05% mercurochrome and the number of oocysts on each midgut were counted. TRA was calculated by the following formula:

1 $${{{\rm{TRA}} = 100 \times \left( {\frac{{{\rm{Mean}}\;{\rm{Oocyst}}\;{\rm{Number}}_{{\rm{neg}}\;{\rm{ctrl}}} - {\rm{Mean}}\;{\rm{Oocyst}}\;{\rm{Number}}_{{\rm{test}}}}}{{{\rm{Mean}}\;{\rm{Oocyst}}\;{\rm{Number}}_{{\rm{neg}}\;{\rm{ctrl}}}}}} \right)}}.$$

where the negative control (neg ctrl) feed used serum from rats immunized with adjuvant only.

SMFAs were performed with heat-inactivated and not heat-inactivated AB + naive human serum pool and rat test sera were heat-inactivated. RBCs from a malaria naive donor and AB + human naive sera for the pool were received from Interstate Blood Bank, Memphis, Tennessee.

### Reporting summary

Further information on research design is available in the Nature Research Reporting Summary linked to this article.

## Supplementary information

Supplemental Material

REPORTING SUMMARY

## Data Availability

Plasmids and other datasets can be provided by N.H.T. upon reasonable request and a completed material transfer agreement. Requests should be submitted to: N.H.T.
