# Supplementary material for: Design of a stabilized non-glycosylated Pfs48/45 antigen enables a potent malaria transmission-blocking nanoparticle vaccine
Source: NPJ Vaccines. 2023 Feb 18;8:20. doi: 10.1038/s41541-023-00619-9 (PMC9938515; doi:10.1038/s41541-023-00619-9)
Supplement: Supplementary file 1 — Supplemental Material [file 41541_2023_619_MOESM1_ESM.pdf]

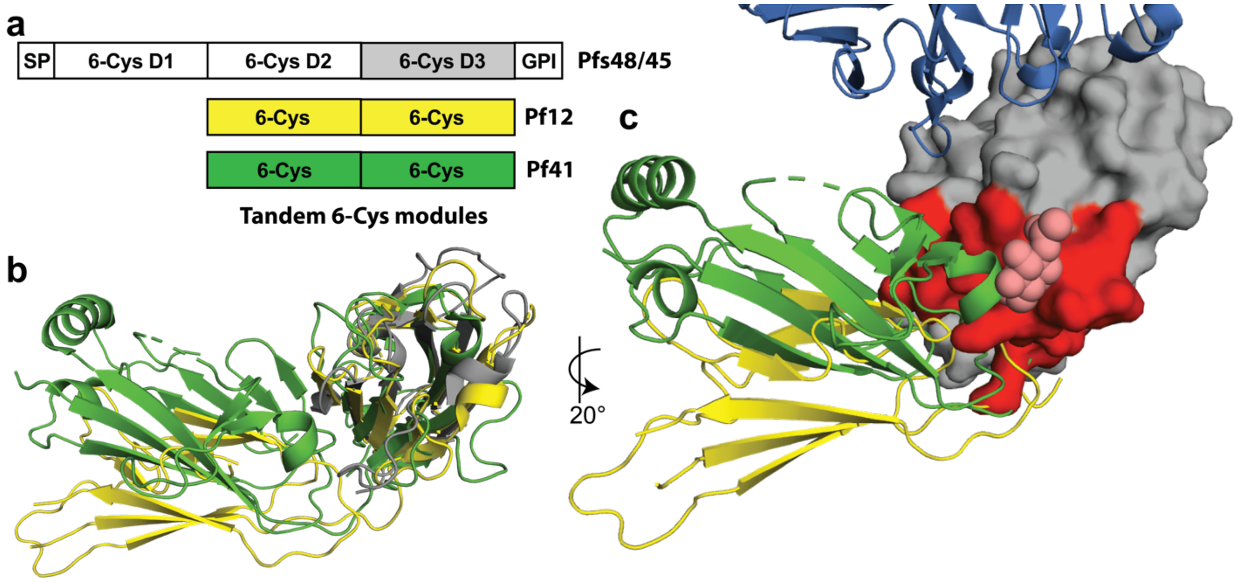

Supplementary Fig. 1. Prediction of the heavily designed residues at the D3/D2 interface. a) Domain architecture of Pfs48/45, Pf12 (yellow), and Pf41 (green) with predicted tandem 6-cys modules aligned. b) The C-terminal 6-cys domains of Pf12 (yellow) and Pf41 (green) were aligned with Pfs48/45 D3 (grey) to predict the location of Pfs48/45 D2. c) Residues of Pfs48/45 D3 (grey) that contact the N-terminal domains of Pf12 (yellow) and Pf41 (green) or NAG303 (pink) are colored red and were heavily designed. Neutralizing mAb 85RF45.1 is shown in blue.

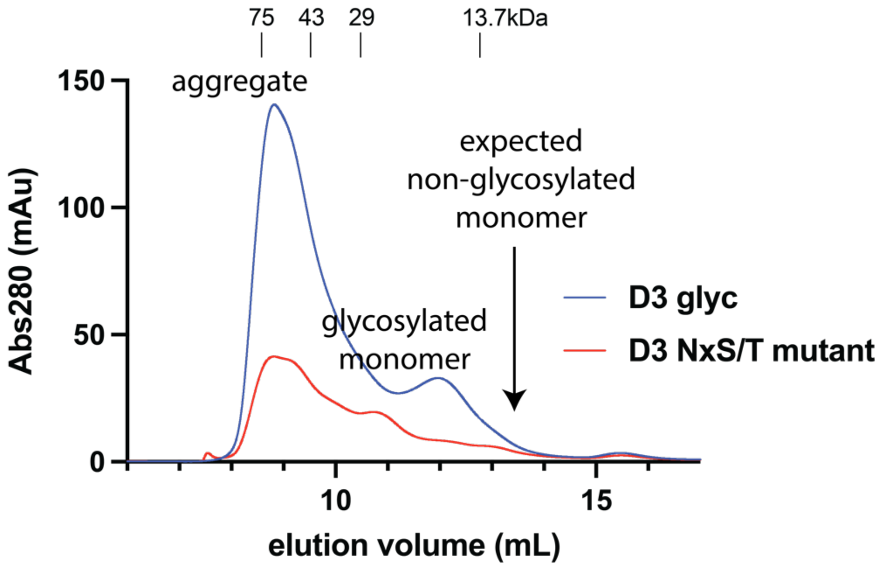

Supplementary Fig. 2. Monomeric WT D3 cannot be purified after mutation of the NxS/T motif. Size-exclusion chromatogram of nickel purified protein.

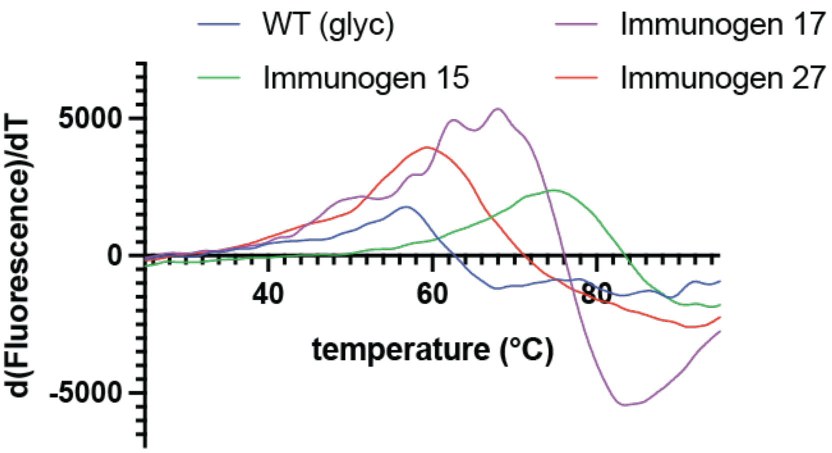

Supplementary Fig. 3. Representative DSF traces of lead immunogens and WT Pfs48/45.

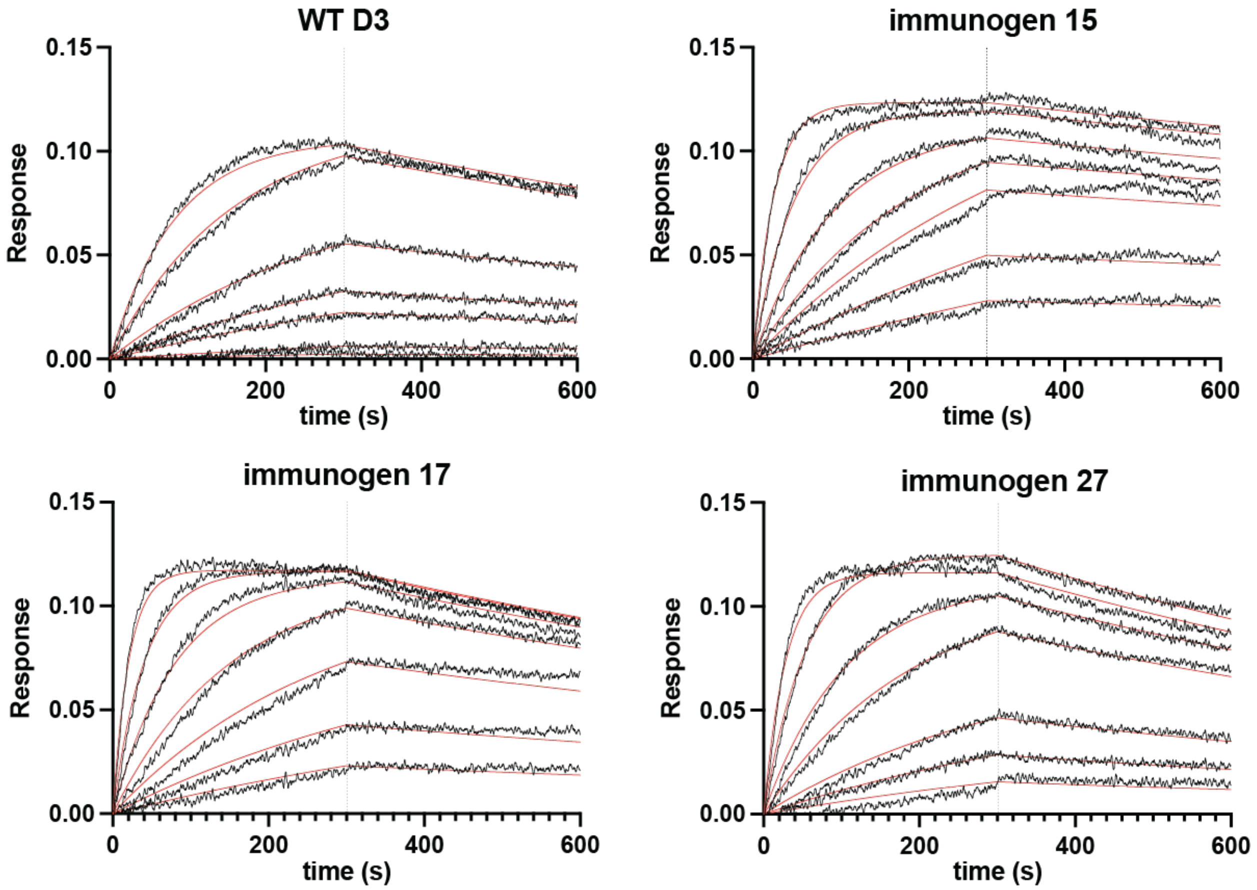

Supplementary Fig. 4. Representative BLI traces of TB31F mAb binding to WT D3 and lead immunogens. Top curves contain 30 nM antigen with a 2-fold dilution series to 0.469 nM.

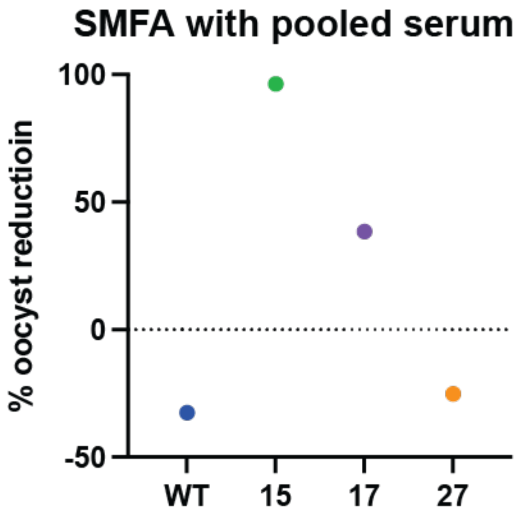

Supplementary Fig. 5. Immunogens 15 and 17 elicit TRA in pooled serum from immunized rats. Dashed line indicates 0% TRA relative to adjuvant only serum.

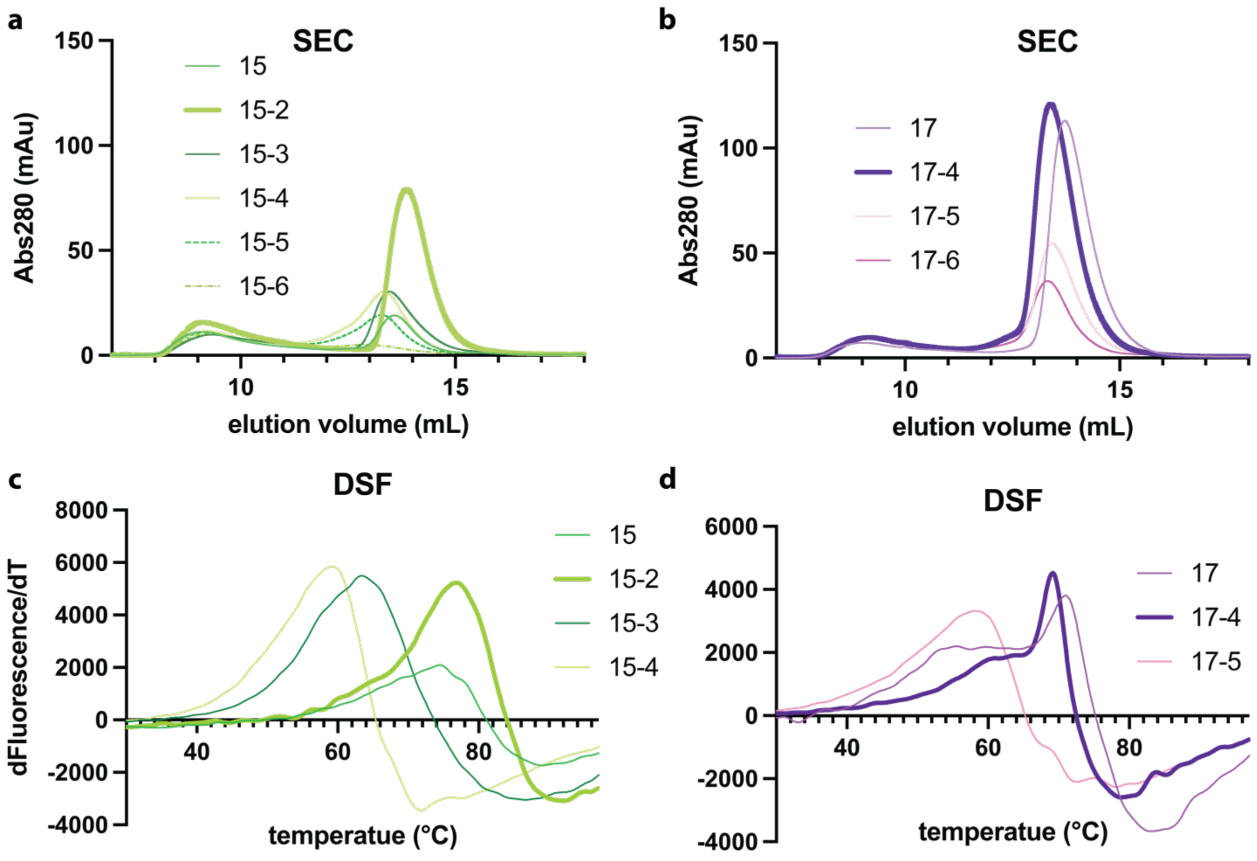

Supplementary Fig. 6. Characterization of optimized immunogens with amino acid reversions to WT. a) Size exclusion chromatogram following Ni purification of immunogens derived from the parent immunogen 15 and b) immunogen 17. c) Differential scanning fluorimetry of the high-yield optimized immunogens based on parent immunogen 15 and d) immunogen 17. Final immuonogens (15-2 and 17-4) are shown in bold.

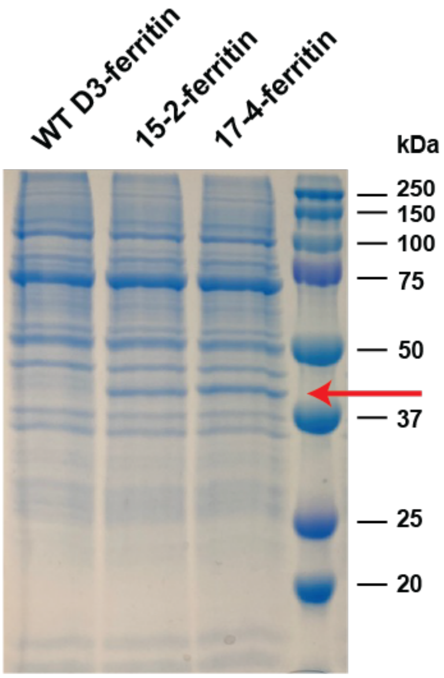

Supplementary Fig. 7. Ferritin fusions of optimized immunogens have much greater expression than WT D3. Cell-free supernatant was analyzed by reducing SDS-PAGE. The red arrow indicates the expected migration of the Pfs48/45-fertritin fusion product.

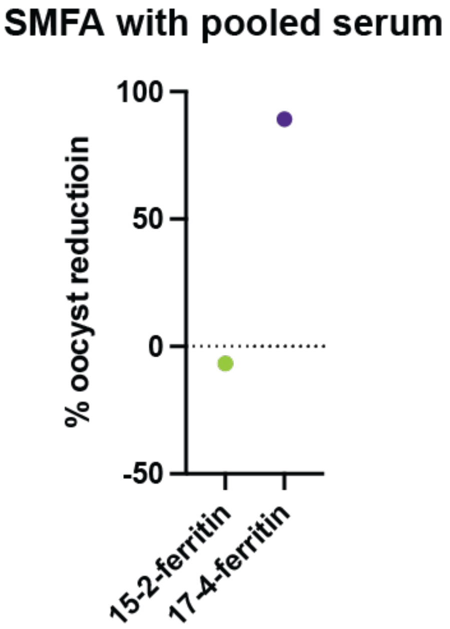

Supplementary Fig. 8. Immunogen 17-4-ferritin elicits high TRA in rats. SMFAs were performed after pooling serum from individual rats. Dashed line indicates 0% TRA relative to adjuvant only serum.

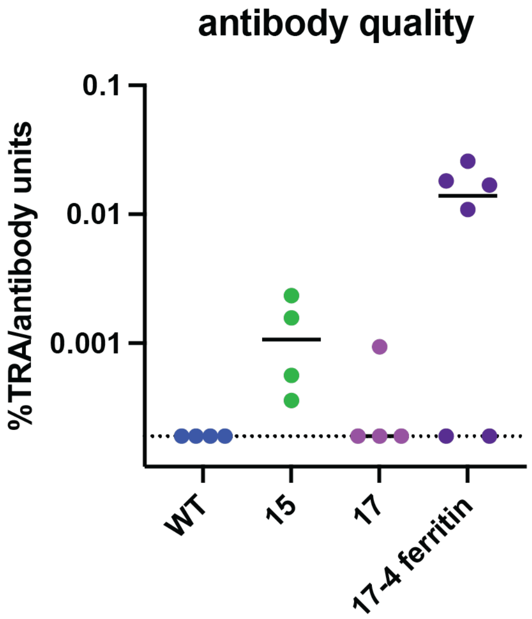

Supplementary Fig. 9. The quality of antibodies improves through the antigen design process. Antibody quality is quantified by normalizing %TRA to the levels of anti-Pfs48/45 IgG in each animal. Animals with negative %TRA values are plotted at the lowest normalized value (dashed line).

```
WT          EKKVIHGCNFSSNVSSKHTFTDSLDISLVDDSAHISCNVHLSEPKYNHLVGLNCPGDIIP  350
15          ..S..A....A..KN..YK.AK.....Q....K.VR.E....H.E...I...........  350
15-2        .....A....A..KA..YK..K..........K..R.E....H.....I...........  350
17          ..S.......A..KN...K.AK.....Q....K.VR.E.E..HKY...I..M........  350
17-4        ..........A...A......K.....................K.......M........  350
6C.mAgE2    .................Y..........................................  350

WT          DCFFQVYQPESEELEPSNIVYLDSQINIGDIEYYEDAEGDDKIKLFGIVGSIPKTTSFTC  410
15          .......HNSA...........ED.L..AN......R.......M.A.I....EP.....  410
15-2        ........NSA...........E..L..A.......R.......M.A.......P.....  410
17          .......HQS.............DV...D.......R.........K.......P.....  410
17-4        ........Q.....................................K.............  410
6C.mAgE2    ..........R...........S.......................L....V........  410

WT          ICKKDKKSAYMTVTIDSAG                                           429
15          .............F...S.                                           429
15-2        .............F.....                                           429
17          .............K...S.                                           429
17-4        ...................                                           429
6C.mAgE2    ...................                                           429
```

Supplementary Fig. 10. Sequence alignment of immunogens designed in this manuscript and an independently derived stabilized immunogen 6C.mAgE2[28].

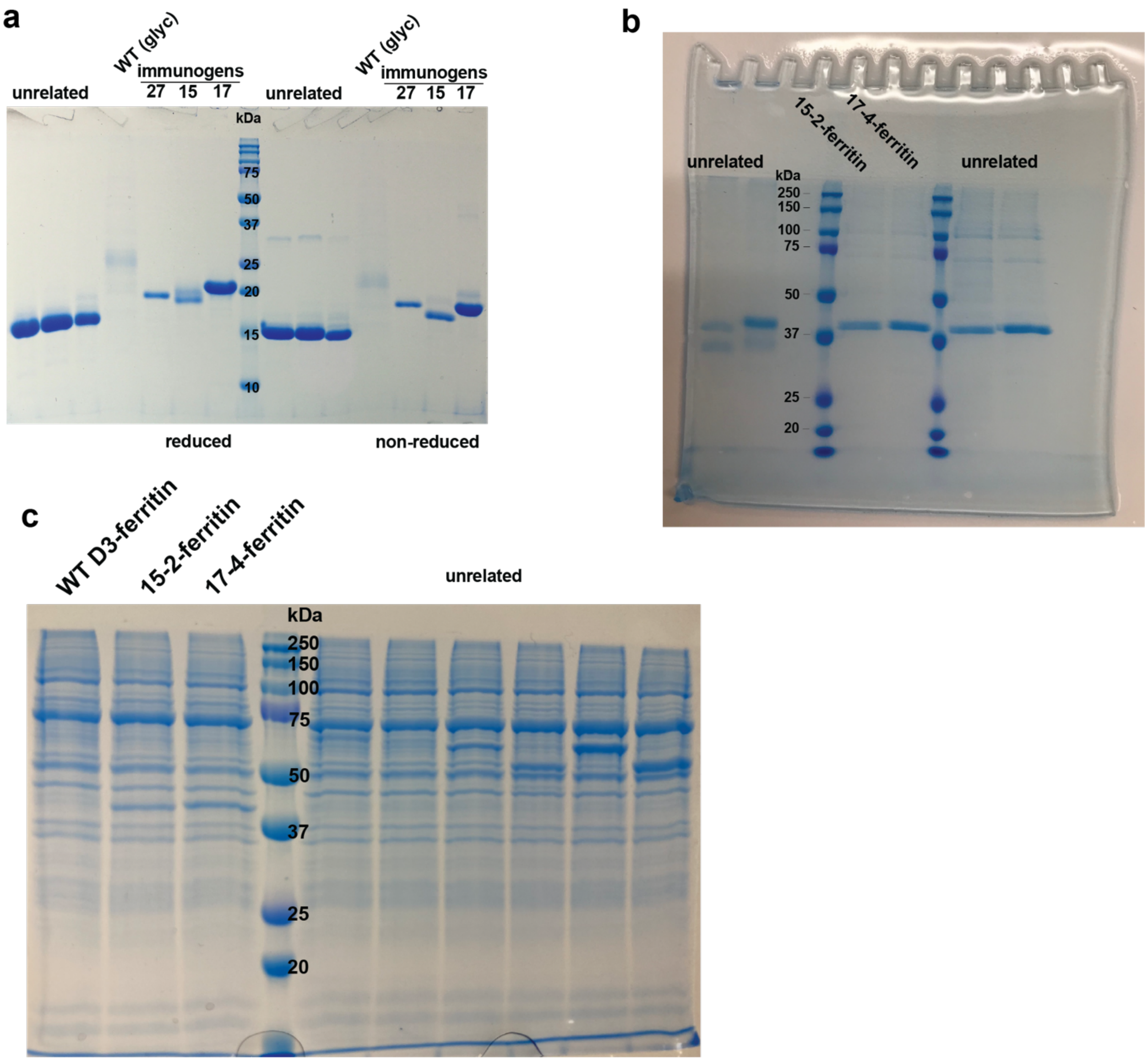

Supplementary Fig. 11. Uncropped gels demonstrating the purity of immunogens and immunogen particles. Gels are related to a) Figure 1e, b) Figure 3c, and c) Figure S7
