# Supplementary material for: Design of a stabilized non-glycosylated Pfs48/45 antigen enables a potent malaria transmission-blocking nanoparticle vaccine
Source: NPJ Vaccines. 2023 Feb 18;8:20. doi: 10.1038/s41541-023-00619-9 (PMC9938515; doi:10.1038/s41541-023-00619-9)
Supplement: Supplementary file 2 — REPORTING SUMMARY [file 41541_2023_619_MOESM2_ESM.pdf]

nature portfolio

Corresponding author(s): Niraj H. Tolia

Last updated by author(s): Nov 23, 2022

Reporting Summary

Nature Portfolio wishes to improve the reproducibility of the work that we publish. This form provides structure for consistency and transparency in reporting. For further information on Nature Portfolio policies, see our Editorial Policies and the Editorial Policy Checklist.

Statistics

For all statistical analyses, confirm that the following items are present in the figure legend, table legend, main text, or Methods section.

n/a	Confirmed	
☐	☒	The exact sample size (*n*) for each experimental group/condition, given as a discrete number and unit of measurement
☒	☐	A statement on whether measurements were taken from distinct samples or whether the same sample was measured repeatedly
☐	☒	The statistical test(s) used AND whether they are one- or two-sided<br>*Only common tests should be described solely by name; describe more complex techniques in the Methods section.*
☒	☐	A description of all covariates tested
☒	☐	A description of any assumptions or corrections, such as tests of normality and adjustment for multiple comparisons
☐	☒	A full description of the statistical parameters including central tendency (e.g. means) or other basic estimates (e.g. regression coefficient) AND variation (e.g. standard deviation) or associated estimates of uncertainty (e.g. confidence intervals)
☐	☒	For null hypothesis testing, the test statistic (e.g. *F*, *t*, *r*) with confidence intervals, effect sizes, degrees of freedom and *P* value noted<br>*Give P values as exact values whenever suitable.*
☒	☐	For Bayesian analysis, information on the choice of priors and Markov chain Monte Carlo settings
☒	☐	For hierarchical and complex designs, identification of the appropriate level for tests and full reporting of outcomes
☒	☐	Estimates of effect sizes (e.g. Cohen's *d*, Pearson's *r*), indicating how they were calculated

Our web collection on statistics for biologists contains articles on many of the points above.

Software and code

Policy information about availability of computer code

Data collection	Unicorn 7, Octet Software v12.0, Gen5 3.08
Data analysis	Prism 9, PyMOL v2.3, ROSETTA 3.10, CD-HIT 2007, Protein Thermal Shift Software v1.4, Octet Data Analysis HT 12.0 Software

For manuscripts utilizing custom algorithms or software that are central to the research but not yet described in published literature, software must be made available to editors and reviewers. We strongly encourage code deposition in a community repository (e.g. GitHub). See the Nature Portfolio guidelines for submitting code & software for further information.

Data

Policy information about availability of data

All manuscripts must include a data availability statement. This statement should provide the following information, where applicable:

- Accession codes, unique identifiers, or web links for publicly available datasets
- A description of any restrictions on data availability
- For clinical datasets or third party data, please ensure that the statement adheres to our policy

Plasmids can be provided by N.H.T. pending scientific review and a completed material transfer agreement. Requests should be submitted to: N.H.T.

Human research participants

Policy information about studies involving human research participants and Sex and Gender in Research.

Reporting on sex and gender	no human research participants
Population characteristics	no human research participants
Recruitment	no human research participants
Ethics oversight	no human research participants

Note that full information on the approval of the study protocol must also be provided in the manuscript.

Field-specific reporting

Please select the one below that is the best fit for your research. If you are not sure, read the appropriate sections before making your selection.

☒ Life sciences ☐ Behavioural & social sciences ☐ Ecological, evolutionary & environmental sciences

For a reference copy of the document with all sections, see nature.com/documents/nr-reporting-summary-flat.pdf

Life sciences study design

All studies must disclose on these points even when the disclosure is negative.

Sample size	Sample sizes were chosen based on animal availability and the ability to see trends in the event of one or two outlier animals. The group sizes are sufficient to support the primary conclusion of the manuscript by showing a statistically significant increase in TRA elicited by the immunogens compared to the native antigen in Figure 4d.
Data exclusions	No data were excluded
Replication	In vitro data were replicated by multiple independent protein purifications and analyses, as indicated in the text. Animal studies were not repeated to conform with proper animal usage guidelines.
Randomization	Animals were randomly assigned to received the indicated immunizations
Blinding	Serum was blinded for SMFAs

Reporting for specific materials, systems and methods

We require information from authors about some types of materials, experimental systems and methods used in many studies. Here, indicate whether each material, system or method listed is relevant to your study. If you are not sure if a list item applies to your research, read the appropriate section before selecting a response.

Materials & experimental systems

n/a	Involved in the study	
☐	☒	Antibodies
☐	☒	Eukaryotic cell lines
☒	☐	Palaeontology and archaeology
☐	☒	Animals and other organisms
☒	☐	Clinical data
☒	☐	Dual use research of concern

Methods

n/a	Involved in the study	
☒	☐	ChIP-seq
☒	☐	Flow cytometry
☒	☐	MRI-based neuroimaging

Antibodies

Antibodies used	peroxidase-conjugated anti-human IgG was added (Jackson ImmunoResearch Laboratories, Inc. Cat. # 109-035-098)<br>peroxidase-conjugated anti-rat IgG (Jackson ImmunoResearch Laboratories, Inc. Cat.#112-035-071)
Validation	TB31F primary antibody was produced as described in the manuscript and validated by measuring binding to WT Pfs48/45 by BLI (see manuscript)

Eukaryotic cell lines

Policy information about cell lines and Sex and Gender in Research

Cell line source(s)	Expi293F cells from ThermoFisher Scientific (cat# A14527)
Authentication	not authenticated
Mycoplasma contamination	not tested for mycoplasma
Commonly misidentified lines (See ICLAC register)	none

Animals and other research organisms

Policy information about studies involving animals; ARRIVE guidelines recommended for reporting animal research, and Sex and Gender in Research

Laboratory animals	9-week old CD1 or Wistar rats
Wild animals	study did not involve wild animals
Reporting on sex	findings apply only to one sex, and sex is not expected to influence the comparative immune response between immunogens and native antigen
Field-collected samples	study did not include samples collected from the wild
Ethics oversight	studies were performed in an AAALAC-accredited facility under the guidelines and approval of the Institutional Animal Care and Use Committee (IACUC)

Note that full information on the approval of the study protocol must also be provided in the manuscript.
